# Configurational Entropy of Folded Proteins and Its Importance for Intrinsically Disordered Proteins

**DOI:** 10.3390/ijms22073420

**Published:** 2021-03-26

**Authors:** Meili Liu, Akshaya K. Das, James Lincoff, Sukanya Sasmal, Sara Y. Cheng, Robert M. Vernon, Julie D. Forman-Kay, Teresa Head-Gordon

**Affiliations:** 1 Department of Chemistry, Beijing Normal University, Beijing 100875, China; meililiu@berkeley.edu; 2 Pitzer Center for Theoretical Chemistry, University of California, Berkeley, CA 94720, USA; akshaya.das@berkeley.edu (A.K.D.); jameslincoff@gmail.com (J.L.); sukanyasasmal@gmail.com (S.S.); sara.y.cheng.20@gmail.com (S.Y.C.); 3 Department of Chemistry, University of California, Berkeley, CA 94720, USA; 4 Department of Chemical and Biomolecular Engineering, University of California, Berkeley, CA 94720, USA; 5 Molecular Medicine Program, Hospital for Sick Children, Toronto, ON M5G 0A4, Canada; vernon.rm@gmail.com (R.M.V.); forman@sickkids.ca (J.D.F.-K.); 6 Department of Biochemistry, University of Toronto, Toronto, ON M5S 1A8, Canada; 7 Department of Bioengineering, University of California, Berkeley, CA 94720, USA

**Keywords:** configurational entropy, force fields, intrinsically disordered proteins

## Abstract

Many pairwise additive force fields are in active use for intrinsically disordered proteins (IDPs) and regions (IDRs), some of which modify energetic terms to improve the description of IDPs/IDRs but are largely in disagreement with solution experiments for the disordered states. This work considers a new direction—the connection to configurational entropy—and how it might change the nature of our understanding of protein force field development to equally well encompass globular proteins, IDRs/IDPs, and disorder-to-order transitions. We have evaluated representative pairwise and many-body protein and water force fields against experimental data on representative IDPs and IDRs, a peptide that undergoes a disorder-to-order transition, for seven globular proteins ranging in size from 130 to 266 amino acids. We find that force fields with the largest statistical fluctuations consistent with the radius of gyration and universal Lindemann values for folded states simultaneously better describe IDPs and IDRs and disorder-to-order transitions. Hence, the crux of what a force field should exhibit to well describe IDRs/IDPs is not just the balance between protein and water energetics but the balance between energetic effects and configurational entropy of folded states of globular proteins.

## 1. Introduction

Intrinsically disordered peptides (IDPs) are a class of proteins that are defined as dynamic structural ensembles rather than a dominant equilibrium structure in solution [1]. Experimental methods such as nuclear magnetic resonance (NMR) spectroscopy [2], single-molecule fluorescence Förster resonance energy transfer (smFRET) [3], and small-angle X-ray scattering (SAXS) [4] can provide restraints on the structural ensemble of IDP systems but are unable to fully resolve important subpopulations of structure relevant for function [5]. Therefore, computational methods play a critical role by first generating putative structural ensembles [6] and secondly reconciling them with the highly averaged experimental information using Monte Carlo optimization [7,8] or, more recently, Bayesian formalisms [9,10,11]. In this work, we are concerned with the generation of IDP ensembles using physically motivated force fields and molecular dynamics simulations (MD) that model protein–protein, protein–water, and water–water interactions at the atomic level.

Nearly all MD simulations of IDP structural ensembles have been generated with pairwise additive force fields that have traditionally been parameterized to reproduce the folded states of proteins [12]. Nonetheless, atomistic force fields have struggled with issues ranging from biases in secondary structure conformations [13,14] or overly structured and collapsed ensembles that do not agree with experimental data on many IDP systems [15,16]. Additionally, IDPs are more solvent-exposed than folded globular proteins, thus the corresponding choice of water model used to simulate IDPs is critical for capturing the correct balance between protein–water and water–water interactions for folded and unfolded states and for disordered proteins [2,17,18]. The D.E. Shaw group was also the first to show that long standard MD simulations—on the order of hundreds of microseconds—are required to ascertain the ability of a force field to maintain the structural integrity of a globular protein [19,20]. We found that similar issues arise for IDPs that also require long simulations and/or accelerated sampling methods to better represent their structural properties [21].

To improve upon MD simulated predictions for IDPs, a few research groups have proposed energy parameter changes to standard force fields to bring them better in line with solution experiments. For the TIP4P-D water model [22], Piana et al. increased the C_6_ dispersion coefficient of the Lennard–Jones parameter by ~50% to make London dispersion interactions more favorable, which, when combined with the Amberff99sb-ildn model [19] for the protein, resulted in more expanded IDPs with an improved agreement with experimental NMR and small-angle X-ray scattering (SAXS) data. Best and Mittal [23] introduced backbone parameter modifications of one of the Amber force fields combined with the TIP4P/2005 water model [24] to reproduce, for example, the temperature dependence of the helix–coil transition for the 15-residue peptide Ac-(AAQAA)_3_-NH_2_. The resulting A03WS/TIP4P/2005 is intended for use for IDPs but, when applied to poly-glutamine IDP in solution, was found to generate mostly featureless and highly extended conformations that do not correctly describe solution experiments [25]. Independently, Henriques et al. have shown that both Amberff99sb-ildn/TIP4P-D and A03WS/TIP4P/2005 reproduce better radius of gyration values for the disordered Histatin 5 (Hst 5) peptide, although both force fields exhibit more turn content for Hst 5 that creates more collapsed states [15]. Robustelli et al. performed extensive millisecond MD simulations on six different pairwise additive protein force fields on a range of fully disordered to folded globular protein systems [20]. These simulations revealed that none of these standard force fields agreed with experimental data for a number of IDP systems while also maintaining the ability to accurately model folded proteins [20].

Therefore, newer protein force fields and water model combinations have been proposed to capture the behavior of IDPs and folded proteins [12]. This is important for at least two reasons. First, they can be used when simulating interactions of IDPs with folded proteins [26], disorder-to-order transitions [27], and folded proteins with intrinsically disordered regions (IDRs) [28]; second, they satisfy the goal of any force field, which is transferability to new protein systems and other emerging problems such as liquid phase separation [29]. An example is the CHARMM36m protein model of Huang et al. that purports to better describe both IDPs and folded proteins using the same set of refined peptide backbone parameters and salt–bridge interactions and an increased Lennard–Jones (LJ) well depth to strengthen protein–water dispersion interactions [30]. These modifications led to a reduction in the percentage of predicted left-handed a-helices, as well as a better agreement with NMR scalar couplings and SAXS curves for folded proteins, although Huang et al. observed that no universal interaction strength parameter in the Lennard–Jones function could generate structural ensembles with good agreement with the experimental radius of gyration measurements for all IDP systems [30].

Hence, the logical next step is to consider more advanced potentials, albeit with a greater computational expense that can be made more accurate by including multipolar electrostatic interactions with many-body polarization that can respond to changes in the solvent conditions around biomolecules [31,32]. One purpose of this study is to ascertain how well the advanced many-body polarizable AMOEBA protein (AmPro13) [33] and water (AmW03) [34] force field performs against experiments across of range of folded proteins, IDRs and IDPs, when compared to a representative standard force field, AMBERff99sb/TIP3P(TIP4p-Ew), and recently modified fixed-charge force fields, CHARMM36(m)/TIP3P(m), where the parentheses refer to alternate protein and/or water model combinations.

The second important purpose of this work is to provide some easily ascertained measures of what constitutes a successful force field that can simultaneously describe both folded proteins and proteins with disorder. We hypothesized that a force field that provides the largest structural deviations and statistical fluctuations, which remains consistent with the experimental Rg of a folded globular protein, will better be able to capture the greater plasticity and match solution experiments for IDPs and IDRs. In fact, we consistently find that the polarizable model better reproduces the experimental Rg [35] for the disordered Hst 5 peptide exhibits a stronger temperature dependence in the disorder-to-order transition for the (AAQAA)_3_ system due to an unusual α−helical structure and maintains a folded core for the TSR4 domain while simultaneously exhibiting regions of disorder. By contrast, the fixed-charge force fields have Rg distributions that are in disagreement with SAXS intensity profiles and contain higher populations of turns for Hst 5 that contribute to a more collapsed state and show little change with temperature for (AAQAA)_3_.

We emphasize that this work is not a quantitative benchmarking paper but to emphasize the qualitative importance of configurational entropy for folded states. By determining a range of metrics for its evaluation such as similarity/dissimilarity and Lindeman criteria, we note that better evidence of fluidity in the folded state will be predictive as to whether a force field will exhibit a better predictive capacity for IDPs/IDRs. This work better places theory as an equal partner to experiment in new areas of IDP studies such as liquid–liquid phase separation that are current and active areas of theory/experimental collaboration.

## 2. Results

The field of biomolecular modeling has historically relied on a simple representation of the potential energy surface of proteins and water based on the pairwise additive approximation of the nonbonded interactions [36].

U_nonbond_ = U_Pauli_ + U_Disp_ + U_Elec_ + U_Pol_ (1)

The U_Pauli_ and U_Disp_ terms are combined within different force fields to formulate a Lennard–Jones 12-6 potential (as is done for Amber and Charmm force fields), whereas the AMOEBA model uses a buffered 14-7 functional form. The U_Elec_ interactions capture classical electrostatics in which Amber and Charmm use partial charges (monopoles), whereas AMOEBA uses a permanent multipole up through quadrupoles. Finally, only AMOEBA contains U_Pol_ for many-body polarization.

**Figure 1:**
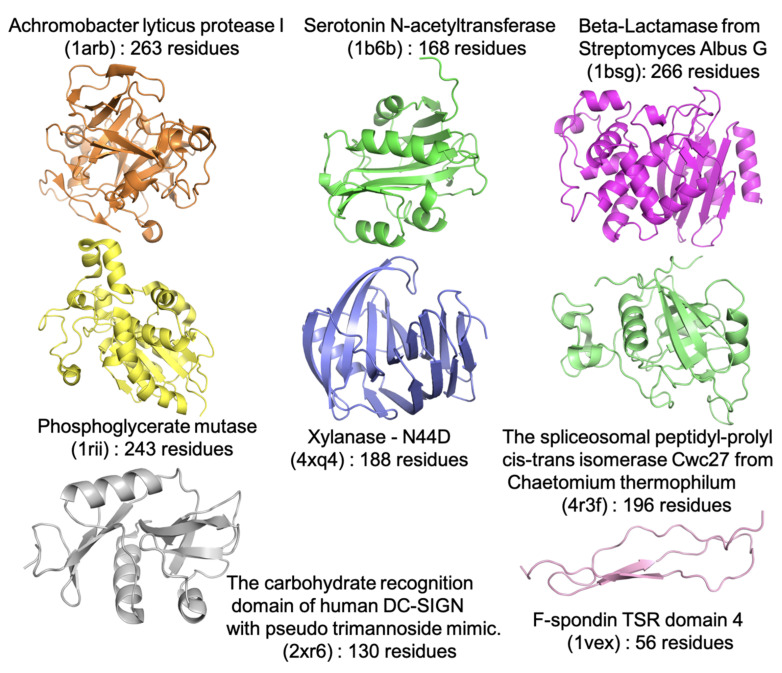
Seven folded proteins (PDB IDs: 1b6b [37], 1arb [38] 1bsg [39], 1rii [40], 2xr6 [41], 4r3f [42], and 4xq4 [43]) and one protein with intrinsically disordered regions (1vex [44]) simulated with polarizable and nonpolarizable force fields.

To compare these force fields for describing the behavior of both folded proteins and IDRs/IDPs, we first consider 7 globular proteins ranging in size from 130 to 266 residues, as shown in Figure 1. These proteins include: a serine protease (1arb), an n-acetyltransferase (1b6b), two hydrolases (beta-lactamase, 1bsg and xylanase, 4xq4), two isomerases (phosphoglycerate mutase, 1rii and cis-trans isomerase Cwc27, 4r3f), the sugar-binding protein DC-SIGN (2xr6), and finally the TSR4 domain (1VEX) as an intermediate class of protein with a small folded core dominated by IDRs.

**Figure 2:**
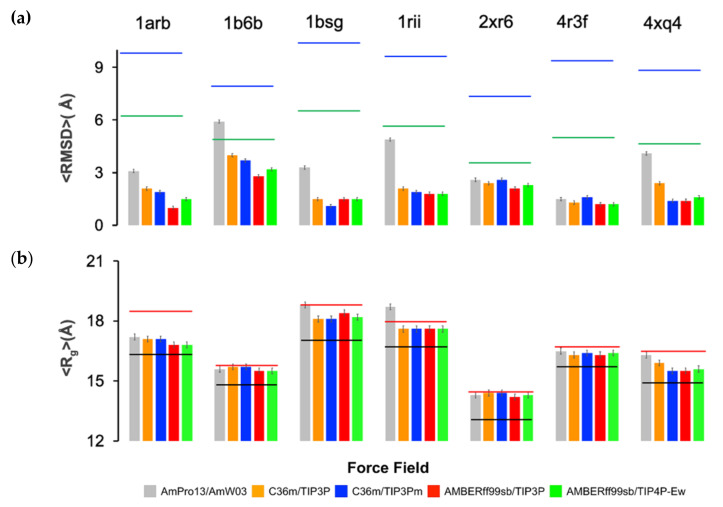
Measures of protein stability when simulated with polarizable and nonpolarizable force fields. (**a**) Root mean square deviation (RMSD) for 1 μs MD simulations for AmPro13/AmW03, C36m/TIP3P, C36m/TIP3Pm, ff99SB/TIP3P, and ff99SB/TIP4P-Ew. The black line is the value of the $D_{0,\;sim}$ metric and the red line the metric and the red line the $D_{0,\;dis}$ metric. (**b**) <Rg> for all force fields and comparison to the Rg of the PDB structure (black) or polymer scaling laws (Supplementary Table S2) as a measure of solution (red). Proteins characterized are 1arb [38] 1b6b [37], 1bsg [39], 1rii [40] 4xq4 [43], 4r3f [42] and 2xr6 [41]

For any biomolecular force field comparison, it is typical to run molecular dynamics simulations of at least ~1 μs to measure protein stability by calculating global metrics such as the root mean square deviation (RMSD) and radius of gyration <Rg> [30]. Figure 2 and Supplementary Figure S1 report on the coordinate RMSD and <Rg> of the seven folded proteins over the 1 μs of MD simulation for each of the force field combinations. All seven folded globular proteins show no evidence of early unfolding events or significant degradation in a secondary structure with any force field, as shown in Supplementary Figure S2 for 1bsg and 1b6b. However, an important distinction is that the polarizable force field exhibits substantially larger root mean square deviations (RMSDs) than those of the nonpolarizable models, although all force fields maintain an average radius of gyration <Rg> in agreement with the experiment.

Although our 1 μs simulation timescales are typical of previous work on measuring protein stability [30], we consider additional metrics for acceptable deviations from the starting structures derived from the PDBs. Figure 2 reports a metric developed by Maiorov and Crippen that provides an empirical relationship to estimate structural similarity $D_{0,\;sim}$ and dissimilarity $D_{0,\;dis}$ for globular proteins (see Supplementary Tables S1 and S2) [45]. Values below or at the similarity measure defines a valid ensemble of structures for which loop regions may reconfigure while not significantly shifting the <Rg> and core fold, while values at or above the $D_{0,\;dis}$ metric distinguish the dissimilarity between a reference structure and its mirror image and thus any large shifts in <Rg> and conformation. In this work, we measure Rg from both the PDB structure for each protein and from polymer scaling law estimates parameterized by PDB structures (see Supplementary Table S2) under poor solvent conditions and structural variations of globular proteins of the same size [46,47]. The larger Rg values from the polymer scaling laws relative to the PDB structure are well within the expectations from solution experiments [48], and consistent with crystal structures differing somewhat from NMR [49] and SAXS [50] ensembles for folded states (Supplementary Tables S1 and S2).

As seen in Figure 2, all force fields yield RMSDs within the range of the $D_{0,\;sim}$ metric for the seven folded proteins. With the exception of 1b6b, for which the <RMSD> using AmPro13/AmW03 is within the $D_{0,\;sim}$ by ~0.5 Å, all models have not fully reached allowed values of the $D_{0,\;sim}$ metric, and no force field exhibits unfolding or instability as measured by $D_{0,\;dis}$ (see Supplementary Figure S2). However, just as importantly, it is also evident that the fixed-charge force fields generally yield folded states with much smaller <RMSD> values, whereas the polarizable force field model is closest to the upper bound of the similarity metric for the globular proteins. In addition, the <Rg> for the pairwise additive models are more often closer to the PDB structure, while the <Rg> values for the polarizable model are more in line with biopolymer scaling law estimates (Supplementary Table S1).

**Figure 3:**
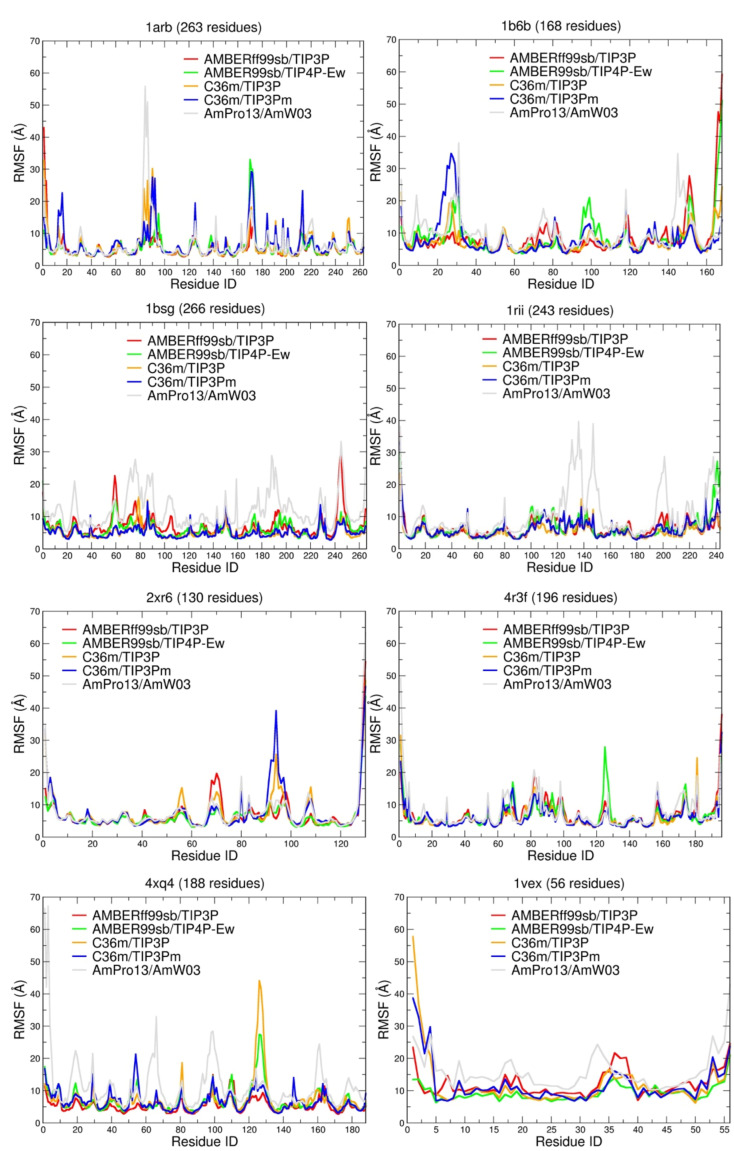
Average root mean square fluctuation for each residue in the simulated trajectories averaged over the last 100 ns. For 1arb [38] 1b6b [37], 1bsg [39], 1rii [40] 4xq4 [43], 4r3f [42] and 2xr6 [41].

Because values of RMSD correlate directly with root mean square fluctuations (RMSF) [51], Figure 3 shows that the <RMSF> by residue for the seven folded proteins is largest on average for the polarizable model relative to the fixed-charge force fields, although large regions of structural stability are evident throughout the structure. The question one might ask is whether the larger <RMSF> by residues of the polarizable model is physically sound and correct, and are the fixed-charge models thus overly rigid?

In order to answer that question, we consider using the Lindemann criterion developed originally for the melting of a solid crystal [52]. The Lindemann value ${∆}_{L}=RMSF/a$ has been adapted to the case of proteins by replacing the crystal lattice constant, a, with an average nonbonded distance [53,54]. Katava et al. provided experimental estimates of the ${∆}_{L}$ from inelastic neutron scattering for hen egg white lysozyme (HEWL) and, assuming a = 4.75 Å, found a Lindemann value at the protein melting temperature ($T_{m}$) of ${∆}_{L}^{exp}\left(T_{m}\right)$~0.17–0.18, driven by the mixing in of a greater proportion of unfolded state fluctuations [54]. Below $\left(T_{m}\right)$, the contributions from unfolded state fluctuations diminish as temperature, of course, decreases, but Zhou et al. showed that the folded-state fluctuations comprise an interior protein core that is suppressed and solid-like (${∆}_{L}^{core}\;$~0.05–0.1) whereas the protein surface is quite fluid (${∆}_{L}^{core}\;$~0.15–0.2) [53,55], which, in part, explains the overall experimental value for the HEWL protein near 300 K of ${∆}_{L}^{exp}\left(300\;K\right)$~0.15–16 in water solvent [54]. Because Katava and coworkers found similar results for myoglobin, crambin, hemoglobin, and BSA, they expect these results to be universal values for any folded state of a globular protein of average size, and hence we rely on comparisons to ${∆}_{L}^{exp}\left(300\;K\right)$ in our simulations of the seven folded proteins analyzed here.

By contrast, the polarizable Table 1 reports the corresponding ${∆}_{L}^{sim}\left(300\;K\right)$ values for each protein, assuming a value a = 4.375 Å, which is an average taken among all previous work [53,54,55], but with the RMSF calculated from the fixed-charge and many-body force field simulations (Figure 3 and Supplementary Table S3). Averaged over all of the folded proteins, the nonpolarizable force fields yield Lindemann values ${∆}_{L}^{sim}\left(300\;K\right)\;$ of ~0.12; to put this value into perspective for the fixed-charge force fields, this value is close to $\sim {∆}_{L}^{exp}\left(230\;K\right)$ for HEWL. By contrast, the polarizable force field predicts <RMSF> values that are ~30% larger than those of the fixed charge models, with values of ${∆}_{L}^{sim}\left(300\;K\right)$~0.16 that are in good agreement with the experimental value at room temperature. Supplementary Table S4 shows that all force fields have a very solid structural core, ${∆}_{L}^{core}\left(300\;K\right)\sim 0.09$ for the fixed charge force fields and ~0.12 for the polarizable model and that their total simulated averages are thus dominated by their surface fluctuations, ${∆}_{L}^{surf}\left(300\;K\right)$, which are largest for the many-body potential (0.155 vs. 0.21). The lower ${∆}_{L}^{sim}\left(300\;K\right)$ values from the fixed-charge force fields are thus indicators that they will generally overestimate the melting temperature and/or the amount of native structure in the unfolded state, an undesirable feature of standard force fields noted previously [54,56,57,58]. From the perspective of the Lindemann criteria, this is because they do not fully activate their allowed thermal vibrations permitted by $D_{0,\;sim}$ in the fully populated folded state, requiring much higher temperatures to exceed the RMSF threshold to realize the larger collective modes for unfolding.

We therefore anticipate that $T_{m}$ values using the polarizable force field will be in better agreement with the experiment because large surface fluctuations are evident by their $D_{0,\;sim}$ values that approach the estimated upper bound [45] while remaining consistent with the folded $R_{g}$. We thus conclude from the folded protein class that force fields should exhibit, in addition to a balance between protein–protein and protein–water energetics, a good balance between energy and configurational entropy in order to realize ${∆}_{L}^{sim}$~$\;{∆}_{L}^{exp}$.

We carry this idea further to predict that the force fields with ${∆}_{L}^{sim}$~$\;{∆}_{L}^{exp}$ for folded proteins will be better suited to representing the structural ensembles of IDRs and IDPs as well; by corollary, force fields with ${∆}_{L}^{sim}$ < ${∆}_{L}^{exp}$ for folded states will not be able to describe the greater plasticity of intrinsically disordered states. To test the extrapolation from folded proteins, we now consider the TSR4 domain (1vex), which comprises a small β-sheet core stabilized by a network of pi-contacts, with large loops that have been classified as intrinsically disordered regions [59]. For TSR4 (1vex), the <RMSD> values for all force fields (Table 2) are well outside the $D_{0,\;sim}$ metric (1.34 Å) and in better agreement with the $D_{0,\;dis}$ value (4.49 Å) given the presence of significant segments of disorder. Figure 3 shows that <RMSF> per residue for TSR4 (1vex) is larger on average relative to the folded protein case for all force fields. For the TSR4 domain, all force fields have a less solid structural core than for the folded proteins, ${∆}_{L}^{core}$~0.16–0.18, and are dominated by large surface fluctuations, ${∆}_{L}^{surf}$~0.18–0.29, that exceed those of the folded proteins. There are no direct-solution experimental data to validate against, but these results support the expectation that the Lindemann criteria value for globular proteins is not universal and cannot be extended to IDRs and IDPs. Even so, we find that the Amber force fields yield the most suppressed ${∆}_{L}^{sim}\left(300\;K\right)$ values, while the C36 and C36m force fields fluctuate more, and the polarizable model yields the largest ${∆}_{L}^{sim}\left(300\;K\right)$ value for the TSR4 domain.

**Figure 4:**
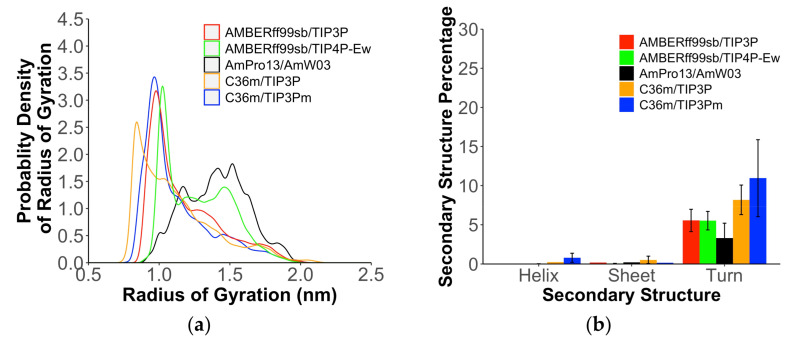
Structural properties for Hst 5 using polarizable and nonpolarizable force fields. (**a**) Probability density estimates of the radius of gyration and (**b**) average percentages of different secondary structures features for the disordered Hst 5 peptide.

These significant ${∆}_{L}^{sim}\left(300\;K\right)$ differences for the TSR4 domain would lead to substantial differences among the force fields with complete disorder. We therefore next consider Histatin 5, a cationic IDP, for which it has been challenging using fixed-charge force fields to achieve agreement with the reported experimental data. These include SAXS form factors that measure a <$R_{g}$> = 13.8 ± 2.2 Å [35] and solution CD and NMR [60,61] measurements, showing that Hst 5 lacks significant secondary structure in aqueous solution, although Hst 5 prefers α-helical conformations in nonaqueous solvents. From Figure 4, we see that the pairwise additive force fields ff99SB/TIP3P, C36m/TIP3Pm, and C36m/TIP3P predict a more narrow $R_{g}$ distribution around compact structures with <$R_{g}$>~10.0–11.0 Å, with higher populations of turns that likely account in part for these collapsed states. The ff99SB/TIP4P-Ew model predicts a bimodal distribution of collapsed and expanded states, but this is in disagreement with the SAXS form factor. The AmPro13/AmW03 potential, with no force field modifications, predicts a more expanded <$R_{g}$>~14.0–14.5 Å in good agreement with the SAXS observable and NMR and CD experiments.

Finally, we consider the very challenging temperature dependence of the (AAQAA)_3_ peptide, in which NMR experiments have previously ascertained a (partial) disorder-to-order transition as the temperature is lowered. There are several issues that are not sufficiently discussed in the literature regarding this peptide and previous simulation attempts to reproduce its behavior. The first is that the NMR experiment was designed to determine the ^13^C-carbonyl shift at each residue, providing an experimental measure of the helicity at each residue for comparison to a helix–coil model that predicts the helicity at each residue [62]. Hence, an overall percentage averaged across all 15 residues is not the correct measure as the NMR shifts are residue-specific values, yielding estimates of 0% to 25%, depending on position, with the N-terminus being more helical. This is in contrast to the highly symmetric prediction of the helix–coil model [62]. Previous studies found that alanine peptides are unusually enriched [63,64] with the π-helix in particular, while the ^13^C-carbonyl chemical shifts are not generally able to differentiate among all three helix categories, especially for fluctuating states. Note that there are statistically different shifts for the stable α−helix and 3_10_ helix [65], suggesting that comparison of structural ensembles to the standard NMR experiment should combine the propensities of the different helix types.

**Figure 5:**
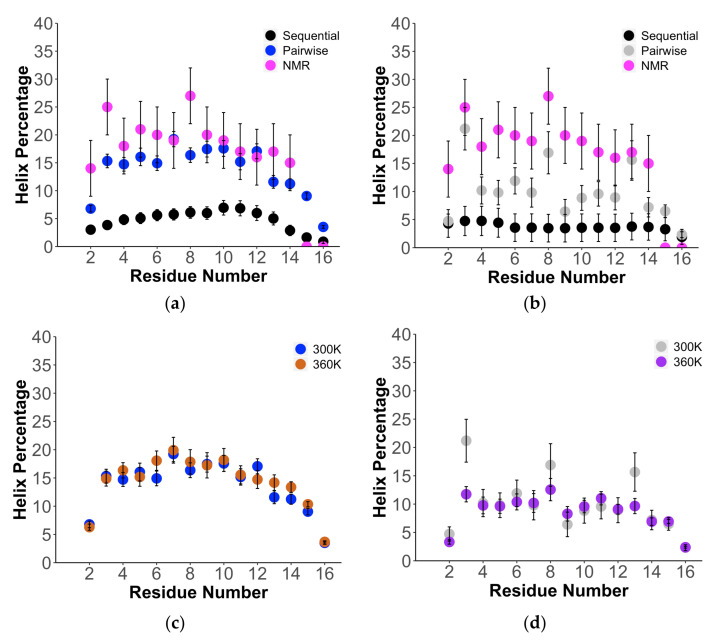
Structural properties for (AAQAA)_3_ using polarizable and nonpolarizable force fields. Comparison of estimated helical propensities from NMR (pink), average α−helix from the simulation assuming 3 sequential residues (black), and pairwise average over any presence of α−helix, π−helix, and 3_10_ helix for (**a**) C36m/TIP3Pm (blue) and (**b**) AmPro13/AmW03 (gray) at 300 K. Comparison of changes in helix propensity with temperature at 300 and 360 K for (**c**) C36m/TIP3Pm and (**d**) AmPro13/AmW03.

We first investigate the definition of an α−helix percentage used by previous research groups, defined as three consecutive residues residing in a broad α−helix basin of the Ramachandran plot (labeled sequential in Figure 5). Unlike most recent studies, we provide individual residue percentages for the (AAQAA)_3_ peptide (Figure 5a,b and Supplementary Figure S3) [66]. As determined by Boostra and coworkers [67], the C36m results depend critically on the “right” water model, i.e., the standard TIP3P water model must be used, to predict the higher helical content at low temperatures, with little helical content observed using TIP3Pm at any temperature. We support that result using TCW sampling in which C36m/TIP3Pm yields ~5% α−helix at 300 K (Figure 5a), as do the other fixed-charge force fields (Supplementary Figure S3), and they all exhibit a flat temperature dependence (Supplementary Table S5) in very good agreement with Robustelli et al. using 20 μs MD simulations [20]. The AmPro13/AmW03 polarizable model gives α−helical percentages that are similar to the Amber and CHARMM force fields for (AAQAA)_3_ peptide, i.e., <~5% with no disorder-to-order transition (Figure 5b and Supplementary Table S5).

Instead, we consider an alternative definition of helical percentages in which the (AAQAA)_3_ peptide might adopt not only α−helix, but π−helix and 3_10_ helix configurations [63] as well based on values of ψ(i) and φ(i+1) values (which we label pairwise in Figure 5). Figure 5a,b and Supplementary Figure S3 show that, when using this definition, the fraction of helical percentages for each residue increases for all force fields and temperatures, ~15–20%, but with important differences between the polarizable and nonpolarizable models. It is seen that the fixed-charge models (Figure 5c and Supplementary Figure S3) have no temperature dependence, with nearly the same helical percentages at 300 and 360 K. By contrast, the AmPro13/AmW03 model shows some temperature dependence, with a loss of helical structure at 360 K relative to 300 K as seen in Figure 5d. This supports our hypothesis that fixed-charge force fields that are overly stabilized for folded proteins will manifest as too inflexible for disordered states, in this case due to the inability to melt the N-terminal helix of (AAQAA)_3_ at high temperatures, unlike the polarizable model, which exhibits a better temperature dependence for the configurational entropy. This result has also addressed a long-standing problem with the characterization of the (AAQAA)_3_ peptide with temperature using simulation that must emphasize not only standard the α−helix the but π−helix and 3_10_ helix categories as well and characterize not average helix percentages over the whole peptide but the residue-by-residue average helical percentage values instead.

## 3. Discussion

We have presented a comparison of a range of pairwise additive force fields and the many-body force field AMOEBA to test their ability to simultaneously describe the stable folded states of seven globular proteins, proteins with regions of disorder illustrated with the TSR4 domain, the Hst 5 IDP, and the partial disorder-to-order transition as the temperature is lowered for the (AAQAA)_3_ peptide. We find that the fixed-charge force fields yield small RMSD differences from the PDB structures of the folded globular proteins, whereas the polarizable model has larger RMSD values that are within the expectations from solution experiments [48,49,50] on folded states. However, we have also shown that force fields that generate the largest RMSDs that are still consistent with the experimental *R_g_*, thus exhibiting larger statistical fluctuations on average, are better able to simultaneously describe the plasticity of proteins with regions of complete structural disorder, as shown for the TSR4 domain, Hst 5, and the (AAQAA)_3_ peptide.

In particular, the polarizable AMOEBA force field presents a significant advantage over a fixed-charge force field for IDP simulations, even those that have been specifically modified to better reproduce IDP behavior, as it does not require any problem-specific parameterization for IDPs and can be used as a general force field for different types of IDPs and their complexes. Our analysis indicates that fixed-charge force fields uniformly describe overly collapsed and rigid structural ensembles of the folded proteins, whereas the polarizable model is inherently more fluid with greater configurational entropy that captures both the folded structure and structural ensembles of IDPs. Finally, we note that other force fields tested previously on (AAQAA)_3_ should be reevaluated to consider both π-helices and 3_10_ helices in addition to the α-helix, with a metric that evaluates the helical content on a residue-by-residue basis as the C-terminal end remains unstructured at any temperature [62] We also note that more current state-of-the-art estimates of helical structure based on NMR shifts could be used to obtain a better experimental reference for this peptide [68,69].

We believe that the analysis we have presented here offers several new ideas on force field validation criteria. The first is to measure the ability of a force field to more systematically approach the full value permitted by the structural similarity $D_{0,\;dis}$ metric for globular proteins [45] , as well as a Lindemann criteria values ${∆}_{L}^{sim}$ that are close to that determined from inelastic neutron scattering experiments and that are touted to be universal criteria for any folded protein in water [54]; a related metric is the ability to reproduce the melting temperature of folded proteins. These measures are best at assessing the balance between energetic effects and configurational entropy and what a force field should exhibit to equally well describe IDRs/IDPs and folded states of globular proteins. While this study has concluded that the polarizable AMOEBA force field is better by these structural and dynamical metrics, it is still an open question as to whether some fixed-charge force fields are capable to the same extent or can be made more capable in this regard. While we found that the pairwise additive force field combinations examined here are not fully sufficient, further evaluation and fitting to reproduce the dynamical criteria introduced can provide good guidance to improving force fields in general.

## 4. Materials and Methods

The Hst 5, TSR4 domain, and the 7 folded protein systems were modeled with the following force field combinations: Amberff99sb (ff99SB) [70] with TIP3P [71] and TIP4P-Ew [72], CHARMM36m (C36m) [30] with TIP3P [71] and Charmm-modified TIP3P (TIP3Pm), and AmPro13 [33] with Amoeba Water03 (AmW03) [34]. We used 1 µs standard MD simulations for the folded proteins, the TSR4 domain, and the Hst 5 system with the OpenMM [73] package for the fixed-charge force fields and the Tinker-OpenMM platform [74] for AMOEBA. We also developed a modified version of the OpenMM [73] and Tinker-OpenMM platforms [74] to perform calculations on graphics processing units (GPUs) with Temperature Cool Walking (TCW) [21,75,76] to further improve the sampling of the (AAQAA)_3_ systems. For (AAQAA)_3_, we considered the force field combinations of ff99sb/TIP4P-Ew, ff99sb-ildn/TIP4P-D, C36m/TIP3Pm, C36/TIP3Pm, and AmPro13/AmW03 models.

### 4.1. System and Simulation Preparation

Initial disordered-state structures for Hst 5 and Ace-(AAQAA)_3_-Nme were generated using the tleap function in the AMBER MD engine [77]. The initial coordinates of the TSR4 and seven folded proteins were taken from their PDB structures. Solvation of these systems were performed using tleap for simulations using the ff99sb force fields, VMD or the online CHARMM-GUI for simulations using the C36m force field [78], and TINKER 8 for simulations using the AmPro13 force field [79]. All simulations were performed on systems with the addition of Na^+^ or Cl^−^ counter-ions to maintain net zero charge.

The Hst 5 system was equilibrated according to the following procedure. First, the fully extended peptide was solvated using a 10 Å buffer, and the system was simulated at 500 K for 1 nanosecond (ns) in the NVT ensemble to collapse the peptide. Second, the peptide was resolvated using a smaller cubic box with side lengths of 59.1 Å, with a total of 6166 water molecules. The resolvated peptide was equilibrated with NVT conditions at 500 K for 1 ns, followed by 1 ns of NVT at 300 K. Finally, the peptide was run in the NPT ensemble at 300 K to equilibrate the size of the simulation box. The initial structure for production NVT MD simulations was chosen based on the maximum probable density.

For the (AAQAA)_3_ system, the peptide we also started from an α-helix and solvated using a 10 Å buffer for the fixed-charge force fields, and the heavy atoms of the protein backbones were harmonically restrained with a spring constant of 10 kcal/mol/Å^2^ during a 1 ns simulation in the NPT ensemble over a temperature range that captures the transition (300, 320, 340, 360, or 380 K). Second, 100 ps of NPT simulations were run where the position restraints of the protein backbone were relaxed from 10.0 to 0.0 kcal/mol/Å^2^, reducing the spring constant by 1.0 kcal/mol/Å^2^ every 10 ps. Finally, 20 ps of NPT simulations were run with no restraints on the protein backbone.

Finally, the larger protein systems were energy minimized to a potential energy tolerance of 0.5 kJ/mol with a nonbonded cutoff of 9.4 Å. The heavy atoms in the protein backbones were harmonically restrained with a spring constant of 10 kcal/mol/Å^2^, and the system was heated in the NVT ensemble from 10 to 300 K at a rate of 1 K/ps using a Langevin integrator with a 1 fs timestep. Once the systems reached 300 K, a 1 ns simulation was run in the NPT ensemble with an rRESPA multi-timestep integrator with a 4 fs timestep for fixed-charge force fields and 2 fs timestep for polarizable force fields, using an Andersen Thermostat at 300 K with a collision frequency of 50 ps^−1^. A Monte Carlo Barostat was used with a target pressure of 1.01325 bar and an exchange attempt frequency made every 50 fs.

### 4.2. Production Simulation Details and Analysis

For the solvated TSR4 and folded proteins, we performed 1 μs molecular dynamics simulations in the NVT ensemble at 300 K with the Bussi thermostat using the RESPA integrator and heavy-hydrogen mass repartitioning with a 3 fs time step. Ewald cutoffs of 7 Å and van der Waals cutoff of 12 Å were used. A pairwise neighbor list for partial-charge and polarizable multipole electrostatics and for van der Waals interactions was used. A grid size of 64 × 64 × 64 Å was used for PME summation and a 10^−4^ Debye convergence criterion for self-consistent induced dipoles. Frames were saved every 10 ps and used to perform further analysis. For (AAQAA)_3_, the TCW simulations were performed in the NVT ensemble with the Andersen Thermostat and velocity verlet integrator with a 2 fs timestep to propagate the target temperature (300, 320, 340, 360, or 380 K) and high-temperature (456 K) walkers. Frames from the low-temperature replica were saved every 1 ps and used to perform further analysis.

Supplementary Figure S1 shows the raw RMSD and RMSF over the 1 μs trajectory for the folded proteins. Analyses of the trajectories were performed using Amber Tools and in-house analysis scripts to analyze the secondary-structure propensity for Hst 5, radius of gyration for Hst 5 and the folded proteins, and/or RMSDs and RMSFs of the protein–water systems using block averaging over ~50 ns blocks over the last 800 ns of the trajectory. For the (AAQAA)_3_ system, a residue was classified as being in a helical conformation using two different definitions when compared with NMR chemical shift data from experiments [62]. The first definition is defined as a series of three consecutive residues where the φ dihedral angle was between −160° and −30° and the ψ angle was between −67° to −7° [64]. The second definition more directly targeted different types of helices; when the first and last residue pairs are excluded, the ψ dihedral angle of one residue and the φ dihedral angle of the next residue sum to −125° ± 10° for the π-helix, −75° ± 10° for the 3_10_ helix, whereas that for the α-helix is −105° ± 10°.

## Figures and Tables

**Table 1:** Lindemann values for 7 folded proteins at 300 K. A value of α = 4.375Å and <RMSF> averaged over all residues (Figure 3, Supplementary Table S2) were used to calculate ${∆}_{L}^{sim}\left(300\;K\right)$.

Force Field/Proteine	${∆}_{L}^{sim}\left(300\;K\right)$							Ave.
	1arb	1b6b	1bsg	1rii	2xr6	4r3f	4xq4	
ff99sb/TIP3P	0.10	0.14	0.14	0.13	0.11	0.11	0.12	0.12
ff99sb/TIP4P-Ew	0.10	0.13	0.12	0.12	0.11	0.12	0.10	0.11
C36m/TIP3P	0.11	0.14	0.11	0.12	0.11	0.12	0.14	0.12
C36m/TIP3Pm	0.12	0.18	0.11	0.13	0.14	0.12	0.12	0.13
AmPro13/AmW03	0.13	0.16	0.18	0.22	0.13	0.16	0.17	0.16

**Table 2:** Fluctuation properties of the TSR4 domain at 300 K. <RMSD> is the average root mean square distance to the starting structure of TSR4. A value of a  = 4.375Å and <RMSF> averaged over all residues of TSR4 were used to calculate the total Lindemann value, ${∆}_{L}^{sim}$. ${∆}_{L}^{core}\;$ was evaluated from the β-sheet core residues; ${∆}_{L}^{surf}$ was calculated from all protein residues not characterized as core residues.

Force Field	$\langle RMSD\rangle$	${∆}_{L}^{core}$	${∆}_{L}^{surf}$	${∆}_{L}^{sim}$
ff99sb/TIP3P	3.8	0.16	0.18	0.17
ff99sb/TIP4P-Ew	3.5	0.16	0.20	0.18
C36m/TIP3P	3.1	0.17	0.23	0.20
C36m/TIP3Pm	3.0	0.18	0.24	0.21
AmPro13/AmW03	5.5	0.20	0.29	0.25

## Data Availability

Data available upon request.

## Supplementary Materials

The following are available online at https://www.mdpi.com/article/10.3390/ijms22073420/s1.
