# Supplementary material for: Configurational Entropy of Folded Proteins and Its Importance for Intrinsically Disordered Proteins
Source: Int J Mol Sci. 2021 Mar 26;22(7):3420. doi: 10.3390/ijms22073420 (PMC8037987; doi:10.3390/ijms22073420)
Supplement: Supplementary file 1 [file ijms-22-03420-s001.pdf]

Supplementary Materials

Configurational Entropy of Folded Proteins and Its Importance for Intrinsically Disordered Proteins

Meili Liu, Akshaya K. Das, James Lincoff, Sukanya Sasmal, Sara Y. Cheng, Robert M. Vernon, Julie D. Forman-Kay and Teresa Head-Gordon

Table S1. Measures of similarity and dissimilarity of folded protein ensembles generated by non-polarizable and polarizable force fields. The similarity/dissimilarity metric and scaling law parameters are reported in Table S2. The <RMSD> and <R_g> are collected over 1 μs simulation timescales. Proteins characterized are 1ARB [1], 1B6B [2], 1BSG [3], 1RII [4] 4XQ4 [5], 4R3F [6], and 2XR6 [7].

**Protein (size)**	**1bsg (266 aa)**	**1arb (263 aa)**	**1rii (243 aa)**	**4r3f (196 aa)**	**4xq4 (188 aa)**	**1b6b (168 aa)**	**2xr6 (130 aa)**
			**Similarity and Dissimilarity Metric (Å)**				
$D_{0,sim}$	6.16	6.11	5.80	5.00	4.86	4.47	3.63
$D_{0,dis}$	10.63	10.58	10.19	9.17	8.98	8.48	7.42
**Force Field/Protein**			$\boldsymbol{< RMSD > \pm < \delta RMSD >}$ (Å)				
ff99sb/TIP3P	1.5 ± 0.28	1.0 ± 0.15	2.1 ± 0.24	1.2 ± 0.13	1.4 ± 0.26	2.8 ± 0.19	2.1 ± 0.30
ff99sb/TIP4P-Ew	1.5 ± 0.16	1.5 ± 0.18	1.9 ± 0.18	1.2 ± 0.20	1.6 ± 0.59	3.2 ± 0.30	2.3 ± 0.27
C36m/TIP3P	1.5 ± 0.16	2.1 ± 0.26	1.8 ± 0.25	1.3 ± 0.17	2.4 ± 0.49	4.0 ± 0.44	2.4 ± 0.33
C36m/TIP3Pm	1.1 ± 0.12	1.9 ± 0.50	1.8 ± 0.23	1.6 ± 0.15	1.4 ± 0.14	3.7 ± 0.56	2.6 ± 0.32
AmPro13/AmW03	3.3 ± 0.77	3.1 ± 0.56	4.9 ± 0.70	1.5 ± 0.39	4.1 ± 0.53	5.9 ± 0.28	2.6 ± 0.20
				$\boldsymbol{R_g}$ **Measures (Å)**			
PDB	17.4	16.5	17.1	15.8	15.1	14.9	13.5
Scaling laws [8,9]	18.9 ± 0.4	18.7 ± 0.4	18.2 ± 0.5	16.8 ± 0.5	16.6 ± 0.5	16.0 ± 0.5	14.6 ± 0.6
**Force Field/Protein**				$\boldsymbol{< R_g >}$ (Å)			
ff99sb/TIP3P	18.4 ± 0.1	16.8 ± 0.0	17.6 ± 0.1	16.3 ± 0.1	15.5 ± 0.1	15.5 ± 0.1	14.2 ± 0.1
ff99sb/TIP4P-Ew	18.2 ± 0.1	16.8 ± 0.1	17.6 ± 0.1	16.4 ± 0.1	15.6 ± 0.2	15.5 ± 0.0	14.3 ± 0.1
C36m/TIP3P	18.1 ± 0.1	17.1 ± 0.1	17.6 ± 0.1	16.3 ± 0.1	15.9 ± 0.1	15.7 ± 0.2	14.4 ± 0.1
C36m/TIP3Pm	18.1 ± 0.6	17.1 ± 0.1	17.6 ± 0.6	16.4 ± 0.1	15.5 ± 0.1	15.7 ± 0.5	14.4 ± 0.3
AmPro13/AmW03	18.8 ± 0.5	17.2 ± 0.1	18.7 ± 0.6	16.5 ± 1.3	16.3 ± 0.1	15.6 ± 0.1	14.3 ± 0.1

Table S2. Scaling Law Relationships used for folded states. The structural similarity $D_{0,sim}$ and dissimilarity $D_{0,dis}$ for globular proteins are from Maiorov and Crippen [10]. The Rg are measured from scaling laws derived over folded proteins in the PDB [8,9].

**Scaling Law**	**a**	**b**	**c**
$D_{0,sim} = a+bN^c$ folded states	−5.74	1.85	1/3
$D_{0,dis} = a+bN^c$ folded states	−4.54	2.36	1/3
$Rg = a + bN^c$ folded states	0.0	2.2	0.38
$Rg = a + bN^c$ folded states	0.0	3.0	1/3

Table S3. Average root mean square fluctuation around average simulated structure (<RMSF>).

	$< RMSF$ (Å) $>$						
**Protein/Force Field**	**1bsg (266 aa)**	**1arb (263 aa)**	**1rii (243 aa)**	**4r3f (196 aa)**	**4xq4 (188 aa)**	**1b6b (168 aa)**	**2xr6 (130 aa)**
ff99sb/TIP3P	0.60	0.43	0.58	0.47	0.54	0.62	0.47
ff99sb/TIP4P-Ew	0.52	0.46	0.54	0.53	0.50	0.58	0.47
C36m/TIP3P	0.47	0.50	0.51	0.53	0.61	0.61	0.49
C36m/TIP3Pm	0.46	0.54	0.55	0.51	0.53	0.80	0.63
AmPro13/AmW03	0.81	0.59	0.95	0.68	0.75	0.70	0.58

Table S4. Lindemann criteria for core and surface residues. Core residues are defined as residues with C-alpha atoms within 0.5*Rg of the center residue in the crystal structure. Surface residues are all protein residues not characterized as core residues. A value of a = 4.375 Å was used to calculate the Lindemann Criteria.

	$\Delta_L^{sim}(300\ K)$ Core							
**Force Field/Protein**	**1bsg**	**1arb**	**1rii**	**4r3f**	**4xq4**	**1b6b**	**2xr6**	**Average**
ff99sb/TIP3P	0.11	0.07	0.10	0.08	0.08	0.09	0.08	0.09
ff99sb/TIP4P-Ew	0.09	0.08	0.09	0.09	0.08	0.09	0.08	0.09
C36m/TIP3P	0.09	0.08	0.08	0.09	0.11	0.10	0.08	0.09
C36m/TIP3Pm	0.09	0.10	0.09	0.08	0.08	0.13	0.09	0.09
AmPro13/AmW03	0.16	0.09	0.17	0.12	0.11	0.11	0.09	0.12
	$\Delta_L^{sim}(300\ K)$ Surface							
**Force Field/Protein**	**1bsg**	**1arb**	**1rii**	**4r3f**	**4xq4**	**1b6b**	**2xr6**	**Average**
ff99sb/TIP3P	0.16	0.12	0.17	0.13	0.16	0.19	0.14	0.15
ff99sb/TIP4P-Ew	0.15	0.13	0.16	0.15	0.13	0.17	0.13	0.15
C36m/TIP3P	0.13	0.15	0.15	0.16	0.17	0.18	0.15	0.16
C36m/TIP3Pm	0.11	0.15	0.16	0.15	0.16	0.17	0.19	0.16
AmPro13/AmW03	0.21	0.18	0.27	0.19	0.23	0.20	0.18	0.21

Table S5. Percentage of α–helix as a function of temperature for the $(AAQAA)_3$ peptide using the sequential definition. The α–helix percentage is defined as 3 consecutive residues within the α–helix basin as described in Methods [11] Aggregate simulation timescales for each non-polarizable force field is 1.0 μs for 300 K and 320 K, and 0.5 μs for 340 K, 360 K, and 380 K. For the polarizable model we simulated for 0.5 μs for all temperatures. All simulations started from a folded α–helix.

Force Field/Temp	300 K	320 K	340 K	360 K	380 K
C36/TIP3Pm	4.98 (0.74)	4.01 (0.51)	3.28 (0.63)	3.72 (0.90)	4.75 (0.81)
C36m/TIP3Pm	4.70 (0.61)	4.11 (0.57)	4.60 (0.80)	4.97 (1.14)	5.31 (1.05)
ff99SB/TIP4P-Ew	3.25 (0.42)	2.64 (0.30)	2.92 (0.44)	2.77 (0.39)	2.69 (0.41)
ff99SB-ildn/TIP4P-D	2.44 (0.65)	3.75 (0.75)	2.80 (0.59)	4.15 (1.03)	2.89 (0.43)
AmPro13/AmW03	3.71 (1.55)	3.32 (1.04)	2.59 (1.59)	1.85 (0.98)	1.12 (0.52)

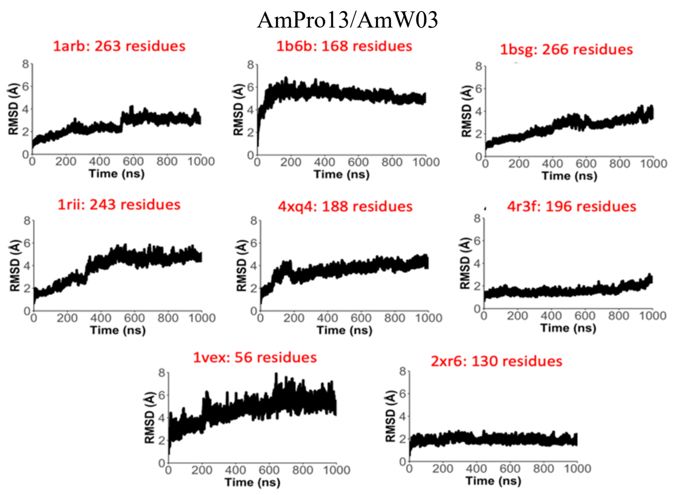

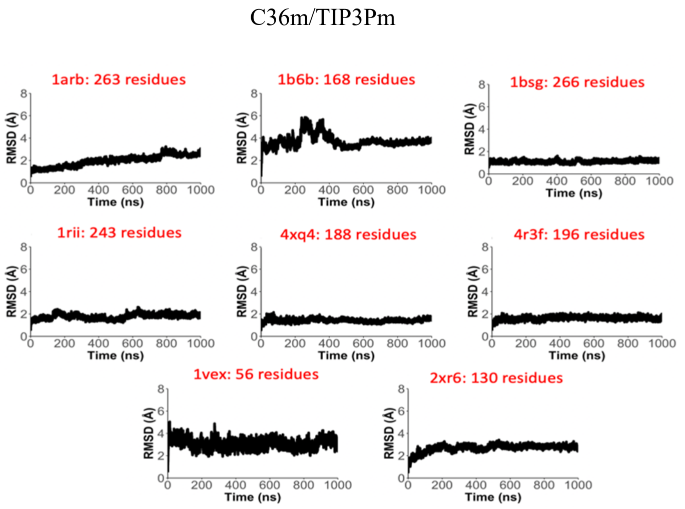

(a) (b)

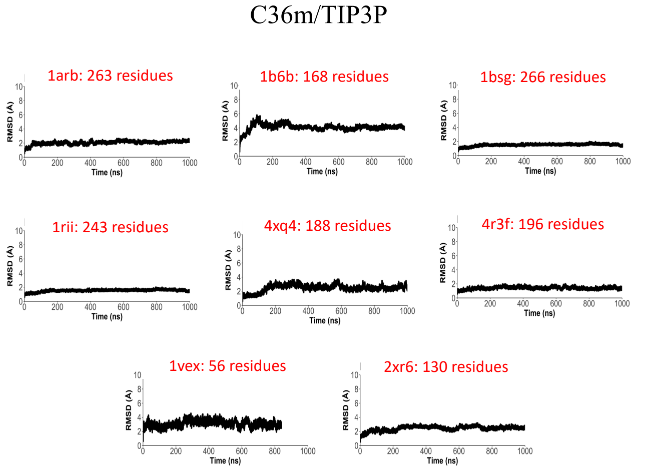

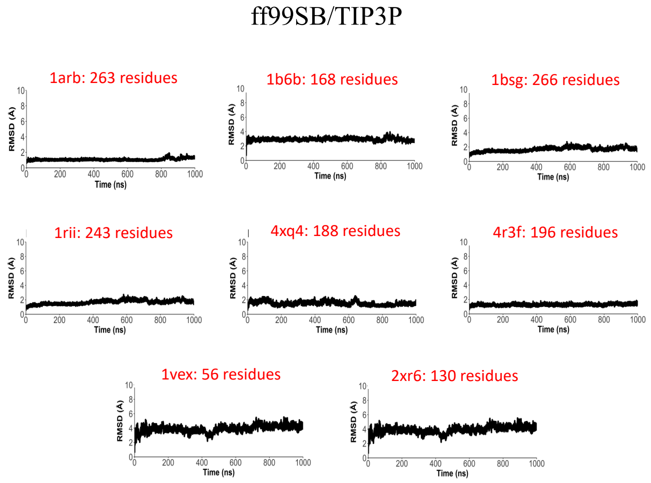

(c) (d)

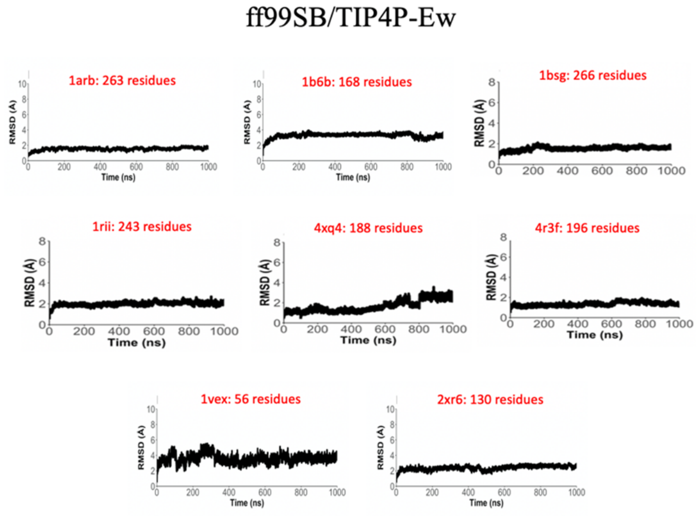

(e)

Figure S1. Protein RMSD when simulated with polarizable and non-polarizable force fields. Root mean square deviation (RMSD) vs. simulation time for 1 μs MD simulations for (**a**) AmPro13/AmW03, (**b**) C36m/TIP3Pm, (**c**) C36m/TIP3P, (**d**) f99SB/TIP3P, (**e**) ff99SB/TIP4P-Ew. Proteins characterized are 1ARB [1], 1B6B [2], 1BSG [3], 1RII [4], 4XQ4 [5], 4R3F [6], 1VEX [12] and 2XR6 [7].

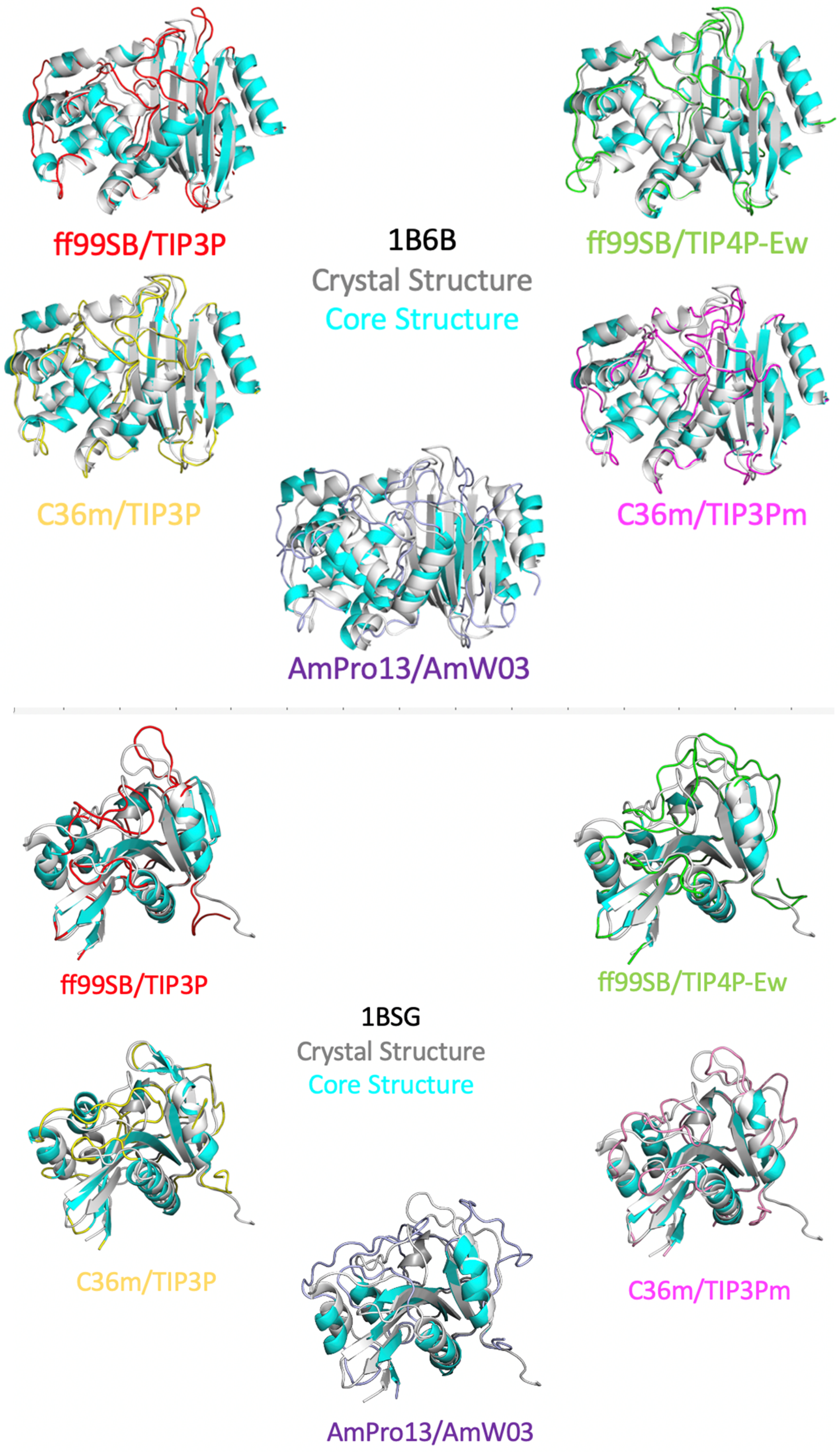

Figure S2. Comparison of core structure and loop restructuring of the simulated force fields against the crystal structure for1B6B [2] and 1BSG [3].

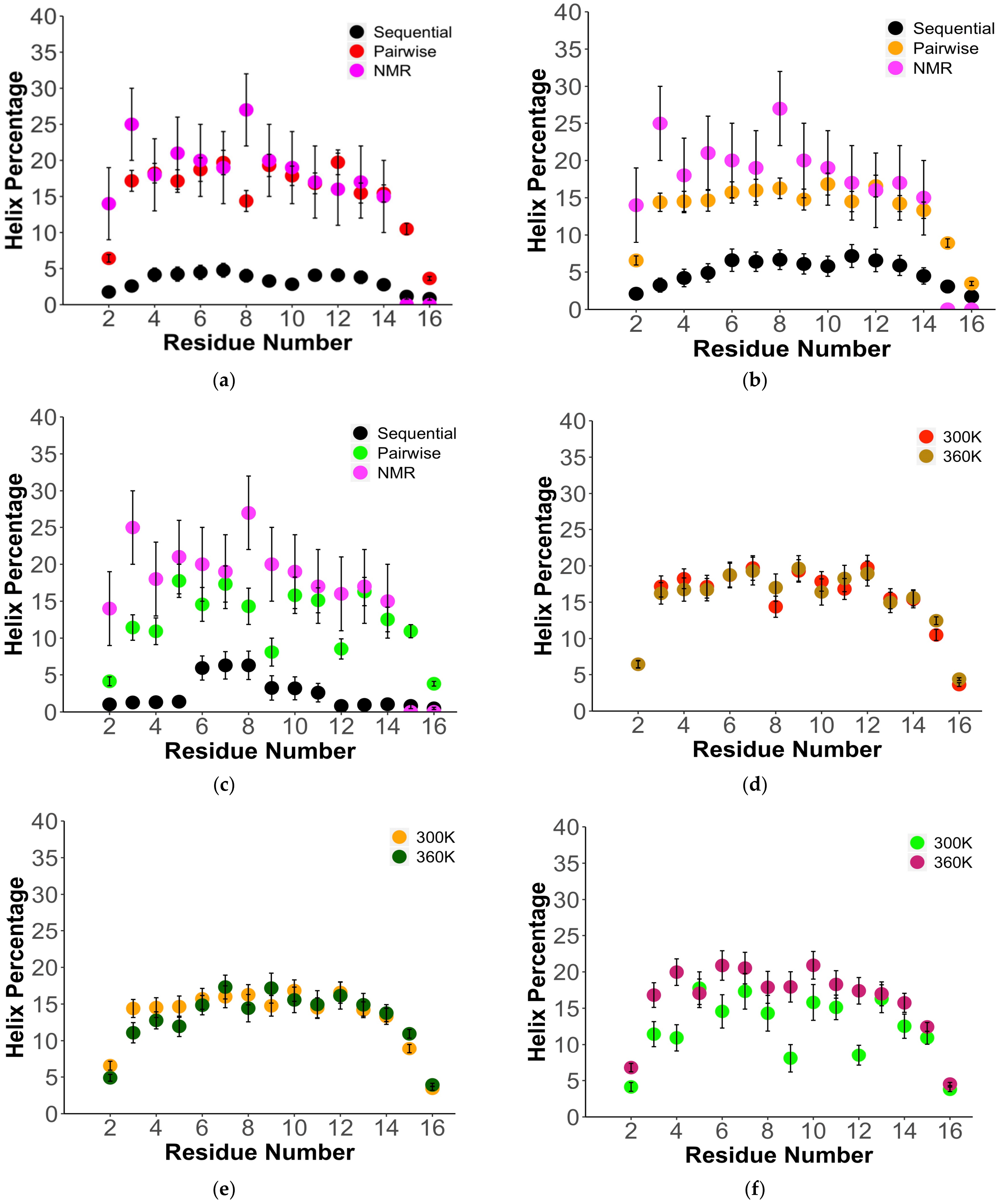

Figure S3. Structural properties for $(AAQAA)_3$ of other non-polarizable force fields. Comparison of estimated helical propensities from simulation assuming 3 sequential residues (black) vs average over any presence of α–helix, π–helix, and 3_{10} helix for all force fields at 300 K compared to NMR estimates (pink). In the SI we consider not only ff99SB/TIP4P-Ew, but other force fields not considered in the main study including C36/TIP3Pm and ff99SB-ildn/TIP4P-D to validate TCW. Comparison of helix definitions for (**a**) ff99SB/TIP4P-Ew (red), (**b**) C36/TIP3Pm (orange), and (**c**) ff99SB-ildn/TIP4P-D (green). Comparison of two temperatures of 300 K and 360 K for average presence of α–helix, π–helix, and 3_{10} helix for (**d**) ff99SB/TIP4P-Ew, (**e**) C36/TIP3Pm, and (**f**) ff99SB-ildn/TIP4P-D. See main text and Figure 4 caption for definitions.

References

1. Tsunasawa, S.; Masaki, T.; Hirose, M.; Soejima, M.; Sakiyama, F. The primary structure and structural characteristics of Achromobacter lyticus protease I, a lysine-specific serine protease. *J. Biol. Chem.* **1989**, *264*, 3832–3839.
2. Hickman, A.B.; Klein, D.C.; Dyda, F. Melatonin Biosynthesis: The Structure of Serotonin N-Acetyltransferase at 2.5 Å Resolution Suggests a Catalytic Mechanism. *Mol. Cell* **1999**, *3*, 23–32.
3. Dideberg, O.; Charlier, P.; Wéry, J.P.; Dehottay, P.; Dusart, J.; Erpicum, T.; Frère, J.M.; Ghuysen, J.M. The crystal structure of the β-lactamase of Streptomyces albus G at 0.3 nm resolution. *Biochem. J.* **1987**, *245*, 911–913.
4. Muller, P.; Sawaya, M.R.; Pashkov, I.; Chan, S.; Nguyen, C.; Wu, Y.; Perry, L.J.; Eisenberg, D. The 1.70 angstroms X-ray crystal structure of Mycobacterium tuberculosis phosphoglycerate mutase. *Acta Crystallogr. D Biol. Crystallogr.* **2005**, *61* (Pt 3), 309–315.
5. Wan, Q.; Parks, J.M.; Hanson, B.L.; Fisher, S.Z.; Ostermann, A.; Schrader, T.E.; Graham, D.E.; Coates, L.; Langan, P.; Kovalevsky, A. Direct determination of protonation states and visualization of hydrogen bonding in a glycoside hydrolase with neutron crystallography. *Proc. Natl. Acad. Sci. USA* **2015**, *112*, 12384–12389.
6. Ulrich, A.; Wahl, M.C. Structure and evolution of the spliceosomal peptidyl-prolyl cis-trans isomerase Cwc27. *Acta Crystallogr. D Biol. Crystallogr.* **2014**, *70* (Pt 12), 3110–3123.
7. Sutkeviciute, I.; Thepaut, M.; Sattin, S.; Berzi, A.; McGeagh, J.; Grudinin, S.; Weiser, J.; Le Roy, A.; Reina, J.J.; Rojo, J.; et al. Unique DC-SIGN clustering activity of a small glycomimetic: A lesson for ligand design. *ACS Chem. Biol.* **2014**, *9*, 1377–1385.
8. Kolinski, A.; Godzik, A.; Skolnick, J. A general method for the prediction of the three dimensional structure and folding pathway of globular proteins: Application to designed helical proteins. *Int. J. Chem. Phys.* **1993**, *98*, 7420–7433.
9. Dima, R.I.; Thirumalai, D. Asymmetry in the Shapes of Folded and Denatured States of Proteins. *J. Phys. Chem. B* **2004**, *108*, 6564–6570.
10. Maiorov, V.N.; Crippen, G.M. Significance of Root-Mean-Square Deviation in Comparing Three-dimensional Structures of Globular Proteins. *J. Mol. Biol.* **1994**, *235*, 625–634.
11. Huang, J.; MacKerell, A.D., Jr. Induction of peptide bond dipoles drives cooperative helix formation in the (AAQAA)3 peptide. *Biophys. J.* **2014,** *107*, 991–997.
12. Paakkonen, K.; Tossavainen, H.; Permi, P.; Rakkolainen, H.; Rauvala, H.; Raulo, E.; Kilpelainen, I.; Guntert, P. Solution structures of the first and fourth TSR domains of F-spondin. *Proteins* **2006**, *64*, 665–672.
